# Sensitivity to and Control of Distraction: Distractor-Entrained Oscillation and Frontoparietal EEG Gamma Synchronization †

**DOI:** 10.3390/brainsci14060609

**Published:** 2024-06-18

**Authors:** Taylor Brown, Kamin Kim, William J. Gehring, Cindy Lustig, Nicolaas I. Bohnen

**Affiliations:** 1 Department of Radiology, University of Michigan, Ann Arbor, MI 48109, USA; browntay@umich.edu; 2 Department of Psychology, University of Michigan, Ann Arbor, MI 48109, USA; um.kaminkim@gmail.com (K.K.); wgehring@umich.edu (W.J.G.); clustig@umich.edu (C.L.); 3 Department of Neurology, University of Michigan, Ann Arbor, MI 48109, USA; 4 Neurology Service and GRECC, VA Ann Arbor Healthcare System, Ann Arbor, MI 48105, USA

**Keywords:** gamma oscillation, EEG, attention, top–down, SSVEP, distractor, PFC

## Abstract

While recent advancements have been made towards a better understanding of the involvement of the prefrontal cortex (PFC) in the context of cognitive control, the exact mechanism is still not fully understood. Successful behavior requires the correct detection of goal-relevant cues and resisting irrelevant distractions. Frontal parietal networks have been implicated as important for maintaining cognitive control in the face of distraction. The present study investigated the role of gamma-band power in distraction resistance and frontoparietal networks, as its increase is linked to cholinergic activity. We examined changes in gamma activity and their relationship to frontoparietal top–down modulation for distractor challenges and to bottom–up distractor processing. Healthy young adults were tested using a modified version of the distractor condition sustained attention task (dSAT) while wearing an EEG. The modified distractor was designed so that oscillatory activities could be entrained to it, and the strength of entrainment was used to assess the degree of distraction. Increased top–down control during the distractor challenge increased gamma power in the left parietal regions rather than the right prefrontal regions predicted from rodent studies. Specifically, left parietal gamma power increased in response to distraction where the amount of this increase was negatively correlated with the neural activity reflecting bottom–up distractor processing in the visual area. Variability in gamma power in right prefrontal regions was associated with increased response time variability during distraction. This may suggest that the right prefrontal region may contribute to the signaling needed for top–down control rather than its implementation.

## 1. Introduction

Brain imaging studies over the past two decades have consistently shown that the top–down control of attention relies on frontoparietal networks [1,2,3]. Recent findings investigate the shift from a modular paradigm of the prefrontal cortex (PFC), assuming that sub-divisions are acting independently, to understanding the dynamic role of the PFC for the coordination of cognitive control [4]. In particular, the right PFC has been consistently identified as an important part of cognitive control networks, although the nature of its contribution is not precisely understood [5,6,7]. Recent efforts have been made to further understand the contributions of the right PFC in cognitive control. Human fMRI studies consistently implicate the right PFC in domain-general cognitive control, specifically as a hub for goal orientation, attentional control, and motivation [8,9]. However, the relationship between right PFC and attentional performance is not entirely straightforward. Increased right PFC activation by populations with impaired top–down control, such as older adults, is sometimes associated with relatively preserved performance, but also with greater impairment [10,11,12,13]. Right PFC activation has been shown to increase with load until it reaches a “crunch point”, after which performance and activation decline [14,15,16]. This performance–activation curve is shifted in older adults where the “crunch point” may occur at lower-level loads due to reaching a “resource ceiling” [10,17,18]. These theories highlight the possibility of an inverted ‘U’ relationship between cognitive load and right PFC activation.

Understanding the modulatory role of the cholinergic system in the PFC may lead to better identification of the underlying mechanisms leading to impairment. Rodent studies indicate that the basal forebrain cholinergic innervation of the PFC region plays an important role in its modulation and visual attention performance, and is highest when the animal is attempting to combat or recover from attentional challenge [11,19,20,21]. Findings from rodent studies have led to the suggestion that rather than reflecting control operations, right PFC activation and acetylcholine increase reflect “attentional effort”, or the motivated recruitment of such mechanisms, which may be implemented by more posterior (e.g., parietal) regions [22,23]. Parallel increases in right PFC activation are seen in human fMRI studies of the distractor condition sustained attention task (dSAT) and transcranial direct current stimulation, which is consistent with the inference that right PFC activation increases have a cholinergic basis and are important for top–down attention [24,25,26]. Although right PFC activation is sensitive to the challenge imposed by the distractor, it may not play a necessary role in maintaining performance in the face of that challenge [25,27,28].

The ability to determine when and where to direct our attention is crucial for filtering large amounts of sensory information. EEG and fMRI studies often implicate the PFC as a modulator for sensory information and important for attention control [9,29,30]. These underlying mechanisms for top–down attention have been tied to changes in alpha and gamma oscillations in the bilateral frontal and right parietal cortex [31]. Increases in EEG gamma-band synchrony may be used as an alternative way of addressing questions of frontoparietal involvement in top–down control and conceptually bridging gaps across rodent micro-dialysis, lesion, electrophysiological studies, and human fMRI research. Studies of local field potentials in animal models and EEG in humans link increases in gamma synchrony to increases in the hemodynamic response thought to underlie the BOLD signal in fMRI [32,33,34].

Neuromodulators, such as acetylcholine, norepinephrine, and dopamine, may also play a role in the ability to resist distractors and enhance cognitive control [19,35,36]. Investigating large-scale oscillatory change in neurotransmission activity utilizing EEG may bridge knowledge gaps between the role of neuromodulators and the frontal and parietal cortex in top–down control. One proposed mechanism for this process is gamma-band synchronization, which is thought to facilitate the functional integration of neural populations forming temporary, large-scale networks, which may be specific for higher cognitive processes such as attention [37,38]. Due to the modulatory role acetylcholine plays in these processes, cholinergic mechanisms may contribute to both bottom–up signal salience and top–down control [20,22,39,40]. Similarly, dopamine modulates executive control in the PFC, and thus, may play a role in goal orientation and working memory [41]. The heightened oscillations in the gamma band are believed to improve the representation of stimuli and the incorporation of stimuli into information processing which may occur through the synchronization of bursts of action potentials, thereby increasing the likelihood of neurotransmitter release [42,43,44].

The present study examines frontoparietal gamma activity in a modified version of the distractor condition sustained attention task (dSAT) [45]. The modified version allows for the dSAT distractor to more clearly become a challenge to top–down attention, while also allowing for EEG measures of the degree to which it captures attention bottom–up. In the present study, we use EEG to examine how low gamma-band (25–55 Hz) oscillations in the frontal and parietal cortex, thought to reflect top–down attention, were related to target detection and changed when attention was challenged by a distracting background. In addition, we used a measure of oscillatory entrainment at 5 Hz to the distractor as an index of the distractor’s ability to capture attention and disrupt goal-driven behavior and examined correlations with the gamma oscillations thought to index control. We measured variations in response time during the distractor and non-distractor condition. Response time, and especially response time variability, can sometimes be a more sensitive measure to attention fluctuations than accuracy and has been suggested to be an intermediate phenotype of attention deficit disorder [46,47]. A participant with good and consistent attentional control would be expected to respond at relatively consistent times across trials, whereas an individual with more attentional fluctuations would have more variability in response times representing a mixture of impulsivity/anticipations, on-task responses, and “just in time” delayed responses. If gamma on a particular trial reflects attentional control on that trial, we should then expect to see a relation between the intrasubject variability in gamma peak dispersion and intrasubject variability in response time.

We tested the following hypotheses. (1) Based on the previous rodent cholinergic and human neuroimaging studies, we expected to find increases in gamma power versus baseline during SAT performance, and that, across subjects, greater gamma power would be associated with better signal detection. (2) To further test the relationship between gamma and detection-related attention, we examined how trial-to-trial gamma variability might be correlated to variability in response time (RT). Participants with fluctuations in attention would also be expected to show fluctuations in gamma, and these would be expected to be further reflected in greater variability in response times. (3) Gamma power, particularly in frontoparietal attentional networks, was expected to increase in response to the distractor and to correlate with distractor-related performance declines. (4) If the distractor induced more attentional fluctuations, we should see an increase in the variability of gamma peak distribution (and RT) in the distractor compared to no distractor condition. (5) Five-Hz oscillations (i.e., the SSVEP) will be observed in the distractor condition. (6) Five-Hz oscillations in the distractor condition, thought to reflect distractor processing, will be greater in the trials in which the target was missed (miss trials) than correctly detected (hit trials) and increases in gamma oscillations in response to the distractor will modulate the magnitude of distractor processing and correlate negatively with the distractor-evoked 5 Hz oscillations. Overall, our hypotheses test the idea that frontoparietal gamma reflects the neural processing involved in the attentional processes that support signal detection, and that increases in these processes support preserved detection in the face of distraction. Part of the methodology and results of this study are from Kim Kamin’s Ph.D. thesis with additional analyses conducted to expand upon the original findings.

## 2. Materials and Methods

### 2.1. Participants

Final analyses included data from 29 healthy young adults (19 females, mean age, 20.1 years, range 18–24 years, 25 right-handed, 1 left-handed, 3 ambidextrous). Participants scored at least 9 on the Extended Range Vocabulary Test (ERVT). Two additional participants were excluded from the analyses, one due to poor performance (below 60% overall accuracy) and the other due to technical errors during the recording. All participants had corrected to normal vision and no history of attention deficit disorder, seizures, migraines, or psychological disorders such as schizophrenia, depression and anxiety.

### 2.2. Ethics

The study was conducted in accordance with the Declaration of Helsinki and approved by the Institutional Review Board (or Ethics Committee) of the University of Michigan (HUM00050064; approval date: 15 June 2011).

### 2.3. Modified Distractor Condition Sustained Attention Task (dSAT)

**Figure 1:**
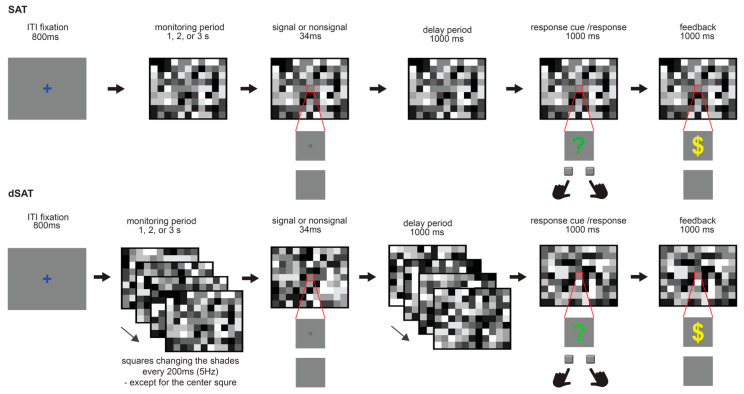
Modified Sustained Attention Task (SAT). Each trial started after an inter-trial interval (ITI) of 800 ms. Participants monitored the center square to detect the presence or absence of a signal that occurred in the middle of that square on a random 50% of trials after 1–3 s of the monitoring period. After a short delay (1000 ms) following the signal/nonsignal presentation, a green question mark appeared in the center square for 1 s as a response cue. Participants reported the presence or absence of the signal by button press using their index fingers (e.g., left for yes, right for no). Correct responses made within the 1 s were followed by reward feedback (a yellow $ sign).

The dSAT is a simple sustained attention task (SAT) in which each trial requires the subject to monitor a central display for an unpredictable amount of time, and at the end of the trial report whether a brief, low-contrast signal did or did not occur. The distractor condition (dSAT) challenges perceptual-attentional performance by rapidly changing the background illumination. In the present study, we used a background consisting of a grid of squares colored in various shades of grey, so that, to the participant, it appeared to be a random assortment of squares and rectangles (Figure 1). In the SAT condition, this background remained stable; in the dSAT condition, the shades of grey at different locations in the grid changed randomly every 200 ms (5 Hz) so that the squares and rectangles seem to either appear, disappear, or move about the screen. Importantly, the distractor flickering at 5 Hz was expected to evoke theta-band (5 Hz) oscillations in visual areas (Steady-State-Visually Evoked-Potential (SSVEP). SSVEP is modulated by selective attention; therefore, the SSVEP in the present paradigm measures the degree to which attention was captured by or misdirected to the distractor [48,49].

Stimuli were presented on a 14-inch CRT screen (800 × 600 screen resolution, 60 Hz refresh rate), using Presentation software (Psychology Software tools; http://www.neurobs.com; Version 16.3 Build 12.20.12). Participants were seated at a 50 cm distance from the monitor in a sound-attenuating, electromagnetically shielded room with dim lighting. Each trial started with a blue fixation (a ‘+’ sign) presented for 800 ms at the center of the screen, followed by a screen divided into 25 by 19 grids, filled with different shades of grey. Each task trial consisted of a variable-duration (1, 2, or 3 s) monitoring period, at the end of which a brief signal (a small grey square, 1 × 1 mm, 34 ms) either did (signal event) or did not (nonsignal event) appear in the center square. After a short delay (1 s), a green ‘?’ sign appeared for 1 s in the center square as a prompt for response. While the response prompt was presented, participants were given 1 s to indicate whether or not they thought a signal occurred on that trial using left and right index finger responses on a standard keyboard (z and/keys on a standard keyboard, respectively, right/left: signal/nonsignal assignment counterbalanced across participants). The 1 s delay between the signal and response cue was inserted to separate the signal-related activity and the response cue-evoked activity. If a correct response was made within the given 1 s, a yellow ‘$’ sign appeared at the center square to notify the participants of the increase in their monetary reward. They were paid 1 cent for each correct percentage and penalized 2 cents for trials where they missed the signal.

The shades of squares in the background grid were controlled in such a way that the net luminance of the whole screen remained constant within and across trials. Seven different shades of grey were used to fill the squares in the grey. The middle darkness grey was assigned to the center square, and the remaining six different shades of grey were equally distributed across the rest of the squares (each shade was assigned to 79 squares) in every grid stimulus.

On standard (SAT) trials, the background remained static throughout the trial; although, to reduce predictability, the distribution of shades across the background grid was unique for each trial. On distractor (dSAT) trials, all the squares in the grid—except for the center square—changed their shades every 200 ms (at 5 Hz) from the beginning of the monitoring period until the onset of the response cue. For both SAT and dSAT trials, the signal was presented on a random half of trials. These SAT and dSAT trials provided the data of primary interest for the present analyses.

To facilitate comparison with event-related fMRI studies [9] using the dSAT we also included filler trials. These started with a grey fixation (rather than the blue fixation used in SAT and dSAT trials) followed by a display that varied in duration, like the SAT and dSAT trials, but did not include the possibility of a signal event or any cue to respond. Instead, participants were told that the grey fixation indicated the start of a rest trial, and that they should simply relax while maintaining fixation on the center square. Paralleling the SAT and dSAT trials, the background was static for half of the filler trials and dynamic for the other half.

Participants were asked to complete 7 blocks, and each block included 72 task trials (36 no distractor, 36 distractor) and 36 filler (18 no distractor, 18 distractor) trials; in total, there were 126 signal and 126 nonsignal trials in each condition.

### 2.4. Procedure

All participants first completed informed consent procedures and a health and demographic questionnaire. The EEG cap and electrodes were set up, and the participants filled in a self-rating scale on everyday attention function utilizing the Imaginal Processes Inventory (IPI) questionnaire [50]. Participants were then given verbal instructions along with a diagram of the stimuli, followed by computerized instructions. The computerized instructions were followed by a practice block in which three mini blocks were embedded. The first mini block consisted of eight consecutive no-distractor (SAT) trials, the second of eight consecutive distractor (dSAT) trials, and the third of 36 trials with all trial types (no distractor (SAT), distractor (dSAT), and filler trials) randomly intermixed. Practice blocks were repeated until the participants reached at least 60% overall accuracy. Participants needed 1–2 practice blocks (1.28 on average). Participants then completed the computerized task, followed by the Edinburgh handedness questionnaire [51], and an eye-test with low contrast Sloan letters (Precision Vision, www.precision-vision.com).

### 2.5. EEG Recording and Preprocessing

Electroencephalography (EEG) was recorded from a 64-channel Ag/AgCl scalp electrodes, two mastoid electrodes, and six electrooculogram (EOG) electrodes, using the BioSemi ActiveTwo system (ActiView version 6.04). The vertical EOG was recorded from electrodes placed above and below each eye, and the horizontal EOG was recorded from electrodes placed external to the outer canthus of each eye. Data points were recorded at a sampling rate of 1024 Hz and referenced to a ground formed by the common mode sense (CMS) active and driven right leg (DRL) passive electrodes (http://www.biosemi.com/faq/cms&drl.htm). To prevent aliasing effects of high-frequency electrode and amplifier noise, low-pass filtering was performed during recording using the decimation filter of the analog-to-digital converter, which has a 5th order sync response with a −3 dB point at approximately 205 Hz (1/5th of the sampling rate (http://www.biosemi.com/faq/adjust_filter_activeone.htm). All electrode offsets were between ±20 mV.

Channels identified visually as noisy during the recording session were replaced using spherical spline interpolation. Across participants, the proportion of channels interpolated was 0.047, with the maximum being 0.123 and the minimum being 0. Data were filtered using an IIR Butterworth bandpass filter (high-pass: 0.1 Hz, low-pass: 70 Hz) and re-referenced by subtracting the average of the two mastoids from the signals of all electrodes. Signals were then visually inspected and screened using the following criteria: blinks at the signal/nonsignal onset, severe noise across the whole channels, unusual sweeps in the mastoid signals, extremely high frequency noise originating from EOG signals. Ocular movement artifacts were corrected using the algorithm from Gratton et al. (1983) [52]. Then, EEG epochs were extracted time-locked to the monitoring period onset with [−750 to 1000] ms time window, baselined to the pre-stimulus period [−750 to 0] ms. Finally, trials (epochs) in which the absolute voltage range exceeded 100 µV for any electrodes were removed from the analysis. Across participants an average of 7.5% of trials were removed. All preprocessing procedures were conducted using EEGLAB (version 9.0.5.6b).

### 2.6. EEG Data Analyses

#### 2.6.1. Local Gamma Oscillation

The time-frequency analysis was conducted using short-time discrete Fourier transform as implemented in the newtimef() function of EEGLAB [53]. The oscillation power was extracted for 30 linearly spaced frequencies between 3 Hz and 60 Hz. The DFT uses sinusoidal wavelets with 3 cycles at the lowest frequency incrementing by 0.5 for higher frequency [53]. Signals preceding the monitoring period ([–400 to –100] ms from the monitoring period onset) were used as the baseline in the time-frequency analyses. As the final assessment of the gamma power surge caused by distractors, we extracted the average power within the gamma frequency band of interest (25–55 Hz), with specific focus on the 25–40 HZ range, a range previously linked with attention [54,55]. The extraction of average power was performed separately for the SAT and dSAT conditions during the 500 ms period following the onset of monitoring ([50–500] ms).

#### 2.6.2. Trial-by-Trial Variations of Gamma Oscillation and Signal Detection Performance

In each individual, the power of oscillations at several gamma-band frequencies was extracted from each trial. The oscillation power was extracted for six linearly spaced frequencies from broadly defined low-range gamma-band (25–55 Hz) using complex Morlet wavelets with 6 cycles. The gamma frequency with the largest power value in a given trial was identified as the gamma peak of that trial. Then, the standard deviation of the gamma peak power values across trials for each individual was used as an estimation of the dispersion of the gamma peak for that individual.

#### 2.6.3. Inter-Trial Coherence on the Distractor-Evoked 5 Hz Oscillations

The distractor-evoked 5 Hz oscillations were evaluated using inter-trial coherence (ITC). Also referred to as “phase-locking factor” or “inter-trial phase coherence”, ITC measures the extent to which the phase-angles of the oscillation at a given frequency are consistent across trials [53,56,57] and is commonly used to estimate oscillations evoked by rhythmic stimuli [58,59]. ITC and SSVEP are complementary measures used to study neural synchronization in response to rhythmic sensory stimulation, particularly in the context of visual processing paradigms. For example, if high SSVEP amplitudes are accompanied by high ITC values, there is evidence of the robust entrainment of neural oscillations to visual stimulus frequency. The measurement value of ITC ranges from 0 to 1, with 0 indicating no coherence and 1 indicating perfect coherence between the EEG data and the time-locking events [53,56]. The newtimef function in EEGLAB was used to obtain the ITC at 5 Hz from each time point in the epoched signal. The average ITC following the onset of monitoring period ([0 to 500] ms) were extracted for the hit and miss trials from each condition. Finally, the significance of distractor-evoked 5 Hz oscillations was assessed using the dSAT-SAT contrast in the hit and miss trials.

### 2.7. Statistical Analysis

Two-tailed paired-sample *t*-tests were used to analyze the behavioral and neural measures in the SAT vs. dSAT conditions. To evaluate the relationships between the behavioral and neural measures, first-level bivariate correlation analyses were used. Influential cases identified by Cook’s distance (Cook’s distance > 4/n, where n is the sample size, 29 in the present study) were excluded from the correlations. Cook’s distance measures the standardized change in the fitted response vector ŷ when the given case is deleted, and conventionally, cases with Cook’s distance greater than 1 or 4/n are considered outliers [60,61,62]. When testing the relationships between the neural and behavioral changes from SAT and dSAT (i.e., distractor effects) residuals were used instead of difference scores. Specifically, linear regression models were conducted on the dSAT measures with the SAT measure as the predictor, and the resulting residuals were used as the variables in the correlation analyses. All statistical analyses were conducted using R (version 3.1.1).

## 3. Results

### 3.1. Behavioral Results

The distractor condition impaired the correct rejection rate (*t*(28) = 7.25, *p* < 0.0005, Cohen’s *d* = 1.35), but enhanced the hit rate (*t*(28) = −5.62, *p* < 0.0005, Cohen’s *d* = 1.04; Table 1). However, response times were slower in dSAT than SAT for both correct rejection and hit trials (correct rejection, *t*(28) = −9.57, *p* < 0.0005, Cohen’s *d* = 1.78; hit, *t*(28) = −10.75, *p* < 0.0005, Cohen’s *d* = 2.00), suggesting the increased hit rate in distractor condition may be driven by a response bias rather than a reduced difficulty of the task. To investigate this possibility, we re-analyzed the data using signal detection theory methods that allow for the determination of sensitivity and bias [63]. Detection sensitivity (d’) was impaired by the distractor (*t*(28) = 4.27, *p* < 0.0005, Cohen’s *d* = 0.79) and importantly, the response bias (beta) differed significantly between SAT and dSAT (*t*(28) = 5.83, *p* < 0.0005, Cohen’s *d* = 1.08), reflecting that participants were guessing ‘yes’ more often in dSAT compared to SAT.

### 3.2. EEG Results

#### 3.2.1. Gamma Power and Variability during SAT Performance (Hypotheses 1 and 2)

Previous rodent and human studies indicate frontoparietal involvement in the signal detection task even without distraction [25,64,65]. We, therefore, began by examining gamma oscillation during the SAT and its correlations with signal sensitivity as indexed by d’. Against hypothesis 1, the average gamma power did not increase during SAT.

**Figure 2:**
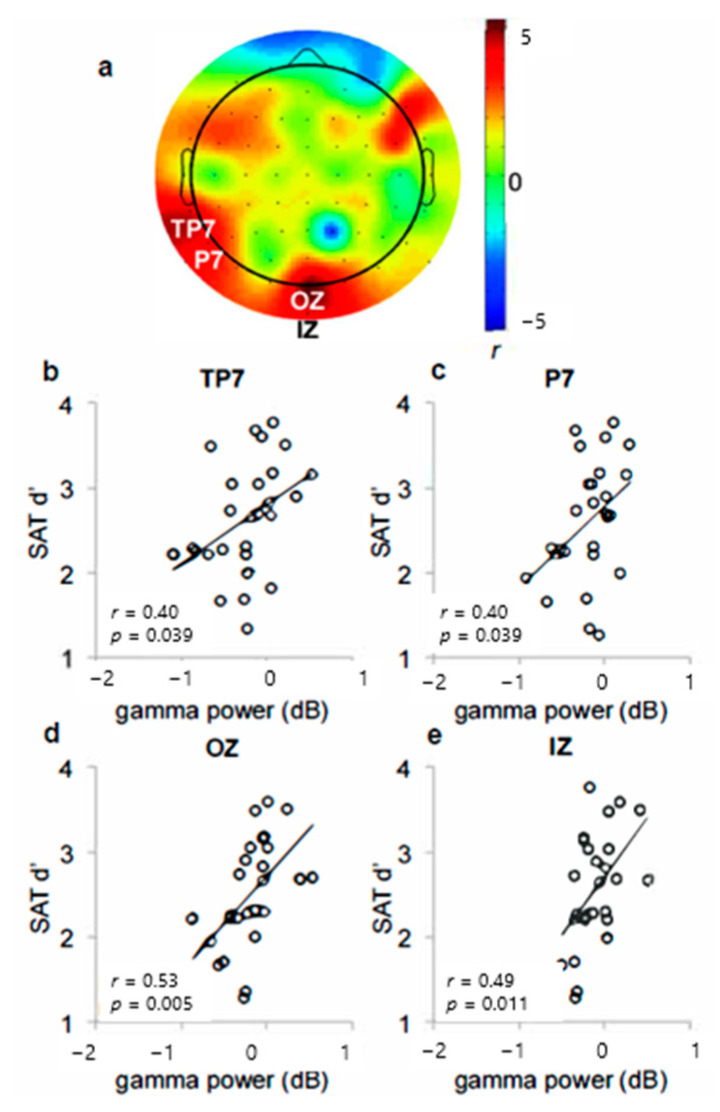
(**a**) Correlations between local gamma power and signal detection sensitivity in the absence of distraction (SAT). The scalp map illustrates the Pearson correlation coefficients at each electrode sites. In two (**b**,**c**) left parietal and two (**d**,**e**) occipital electrode sites, greater local gamma power was associated with better signal detection sensitivity.

**Figure 3:**
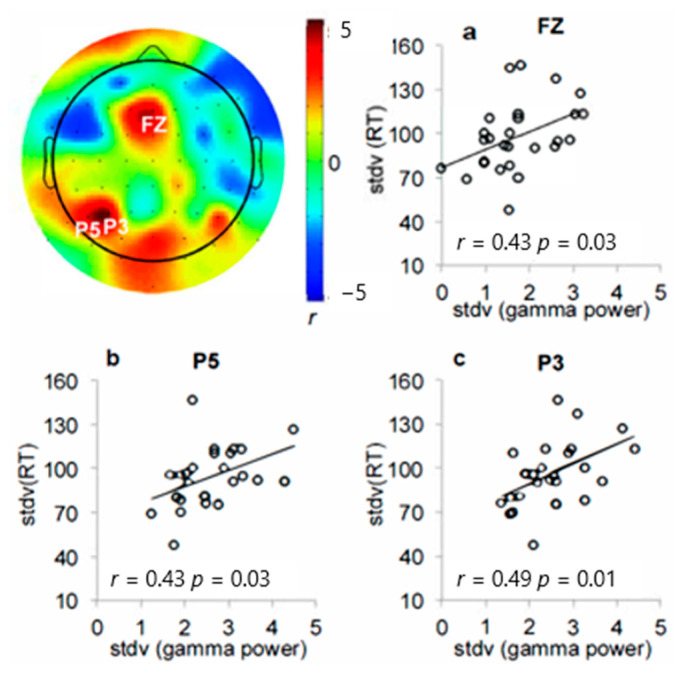
Correlations between the trial-by-trial variation of local gamma peak and response time variation. Greater variations of gamma peak across trials in the (**a**) mid-frontal and (**b**,**c**) left parietal electrode sites were associated with greater fluctuation of response time.

Although mean gamma power measured across subjects did not increase, examination of the individual differences data showed that greater gamma power in the left temporoparietal (TP7, P7) (Figure 2b,c) and occipital (OZ, IZ) (Figure 2d,e) electrodes was significantly associated with better signal detection sensitivity (Figure 2a). These correlations were unique to the SAT condition except for electrode P7 (dSAT *p*s > 0.1 except for P7; further discussed below). The right PFC correlation fell short of standard thresholds for statistical significance (*r* = 0.36, *p* = 0.07), but may still be of conceptual interest because of the previous studies from both rodents and humans, indicating right PFC involvement in SAT performance [25,64,65]. Additionally, there was a significant relationship between the intrasubject variability in gamma peak dispersion and intrasubject variability in response time (Figure 3). The dispersion of the gamma peaks across trials was significantly correlated with greater RT variance in the midline frontal and left parietal electrodes (Fz, P3, P5, *p*s < 0.05).

#### 3.2.2. Changes in Gamma Power and Variability Related to Distraction (Hypotheses 3 and 4)

**Figure 4:**
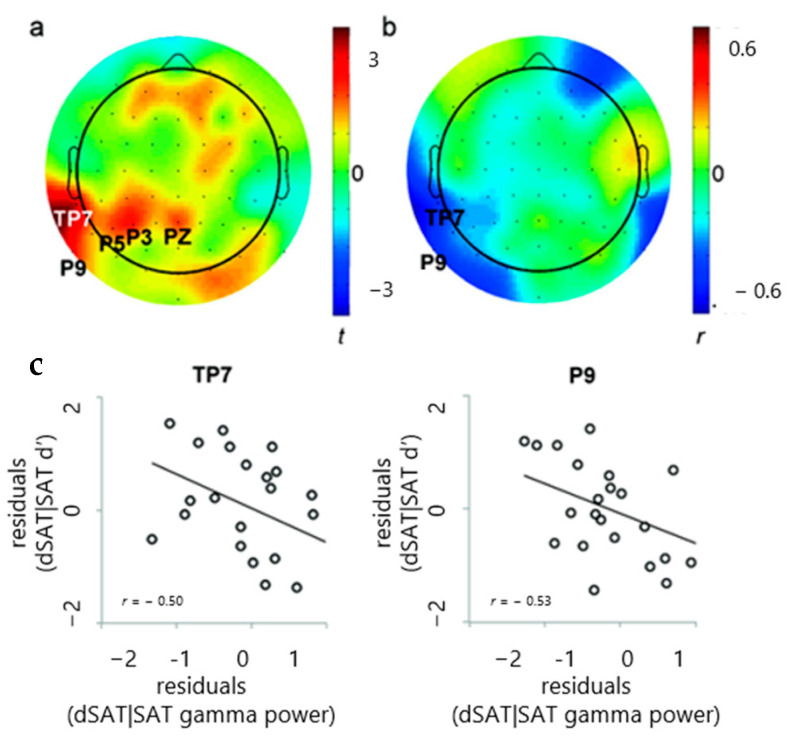
(**a**) Gamma increases in response to distraction depicted from the *t*-values for gamma oscillation power from dSAT and SAT (dSAT-SAT). Gamma power significantly increased in response to distraction in the left parietal electrode sites. (**b**) Correlations between the gamma increases in response to distraction and preserved signal detection sensitivity. (**c**) In the two far lateral electrode sites (TP7, P9) with significant gamma increases in dSAT, greater gamma increases were associated with greater distractor-related declines.

Significant increases in gamma power in response to distraction were observed in five left parietal electrodes (Figure 4a, P3, *t*(28) = 2.39, *p* = 0.02, Cohen’s *d* = 0.44; P5, *t*(28) = 2.05, *p* = 0.049; Cohen’s *d* = 0.38; P9, *t*(28) = 2.64, *p* = 0.013, Cohen’s *d* = 0.49; PZ, *t*(28) = 2.13, *p* = 0.04, Cohen’s *d* = 0.40; TP7, *t*(28) = 3.20, *p* = 0.003, Cohen’s *d* = 0.59; *p* ≥ 0.1, Cohen’s *d* < 0.33 in all other electrodes).

We next examined the correlations between the neural and behavioral distractor effects in these electrodes. Residual scores for dSAT|SAT were used rather than simple difference scores, where dSAT|SAT = dSAT gamma power—SAT gamma power (increase or decrease in dSAT gamma power greater than predicted by SAT). This choice was made because the latter tend to be less reliable and more susceptible to baseline differences. Among the five electrodes that exhibited significant gamma increases in response to distraction, two left parietal electrodes (TP7 and P9) showed significant correlations between changes in gamma power and changes in signal detection sensitivity, |*r*| > 0.5, *p* < 0.01 in both electrodes (Figure 4b). Participants who showed a greater increase in gamma had greater distractor-related performance declines, suggesting that the detection performance may be increasingly impaired in participants with greater gamma increases.

**Figure 5:**
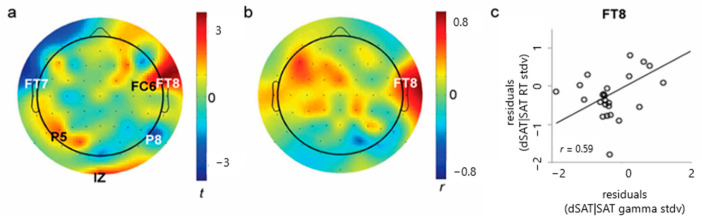
(**a**) Gamma power variability increases in response to distraction. In the right frontal, left parietal and occipital electrode sites (FT8, FC6, P5, Iz), gamma peaks were significantly more dispersed in dSAT compared in SAT. *p* < 0.05 in FC6, FT7, P5, P8, IZ, *p* < 0.001 in FT8 (**b**) Distractor-related gamma dispersion and response time variation. (**c**) The gamma dispersion increase in response to distraction was associated with greater increase in the response time variation in the right frontal electrode (FT8).

The negative correlation between parietal gamma increases and the size of the distractor effect contrasts sharply with the correlation pattern seen for increases in right prefrontal gamma variability (Figure 5). Gamma peak variation increased significantly in response to distraction in the right frontal (FC6, FT8), left parietal (P5), and occipital (Iz) electrodes (Figure 5a, FC6, *t*(28) = 2.36, *p* = 0.03, Cohen’s *d* = 0.44; FT8, *t*(28) = 3.87, *p* = 0.0006; Cohen’s *d* = 0.72; P5, *t*(28) = 2.10, *p* = 0.045, Cohen’s *d* = 0.39; Iz, *t*(28) = 2.23, *p* = 0.03, Cohen’s *d* = 0.41). Consistent with the hypothesis that right prefrontal gamma variance indexes fluctuations in top–down control, the right frontal electrode (FT8) showed significant correlations between changes in gamma peak dispersion and changes in response time variation, *r* = 0.58, *p* = 0.001; Figure 5b,c). Participants who showed a greater increase in right prefrontal gamma dispersion in response to distraction had greater distractor-related response time fluctuation.

#### 3.2.3. Distractor-Entrained Oscillation: Inter-Trial Coherence (ITC) (Hypotheses 5 and 6)

**Figure 6:**
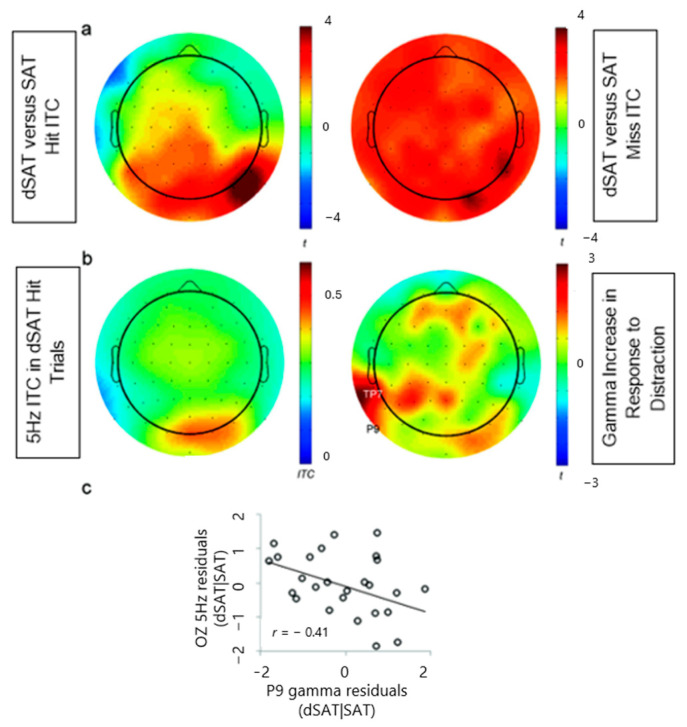
(**a**) Scalpmaps showing *t*-values from a comparison of of 5 Hz ITC in dSAT vs. SAT in hit (left) and miss (right) trials. The distractor with periphery peripheral visual changes at 5 Hz evoked significant 5 Hz ITC in the occipital electrode sites for both hit and miss trials. This effect was prominently more robust and global in miss trials. (**b**) The scalpmap of 5 Hz ITC in dSAT hit trials (left). The right panel highlights left parietal electrode sites with significant gamma increase in response to distraction associated with preserved signal detection sensitivity (right, Figure 4a) (**c**) In trials where targets were correctly detected (hit trials) in the presence of distraction (dSAT), greater local gamma power in the left parietal electrode site (P9) was associated with smaller distractor-evoked 5 Hz oscillations in dSAT.

The scalpmap in Figure 6a depicts the *t*-values resulting from dSAT vs. SAT paired-sample *t*-tests on the 5 Hz ITC separately for the hit (left) and miss (right) trials. The 5 Hz distractor evoked significant 5 Hz ITC in parietal and occipital regions in the hit trials (Figure 6a, scalpmap on the left; *p* < 0.05 in OZ, O1, O2, POZ, PO4, PO8, P2, P6, P7, P8, P10). Importantly, the distractor-evoked ITC at 5 Hz was dramatically more robust and global in miss trials (Figure 6a, scalpmap on the right; *p* < 0.05 except for the following 10 electrode sites: FZ, AF4, CP2, CP5, P10 (0.05 ≤ *p* ≤ 0.06), FT8, FC6, C4, P9, IZ (0.07 ≤ *p*). This pattern is consistent with hypothesis 5 where 5 Hz oscillations are present in the distractor condition and misses may, in many cases, have occurred because participants’ attention was occupied by the distractor. 

The distractor-evoked ITC may reflect attention to the distractor, and gamma modulations reflect cognitive control employed to resist the distractor, then distractor-evoked ITC may be modulated by gamma oscillations in the attentional network. For this analysis, we chose electrode sites of interest for the top–down modulatory and bottom–up distractor processing and tested the dynamics between the two in dSAT hit trials. We selected TP7 and P9 as the electrodes of interest for the top–down modulatory oscillations because their gamma oscillations significantly increased in response to distraction and these increases were associated with preserved signal detection performance (Figure 6b, scalp map on the right; also see section Gamma increase in response to distraction (also shown in Figure 4a). Three occipital electrode sites (O1, OZ, O2) were selected for the bottom–up distractor-evoked 5 Hz oscillations because those exhibited the most prominent distractor-evoked 5 Hz ITC in the hit trials (Figure 6b, scalp map on the left). We then examined the correlations between the distractor-related increases of gamma power in the top–down modulatory sites and the 5 Hz ITC in the bottom–up distractor processing electrode sites (i.e., dSAT|SAT residuals). Greater distractor-induced increases of gamma power in the left parietal electrode site P9 were significantly associated with smaller distractor-evoked 5 Hz ITC in the occipital site OZ (Figure 6c, *r* = −0.41, *p* = 0.04; other p’s > 0.3).

## 4. Discussion

As predicted, the present study found correlations between gamma and signal detection sensitivity (Figure 2), and correlations between intrasubject variability in gamma peak and response times (Figure 3). Additionally, the study found that distractor-related increases in gamma variability and response time variability were related (Figure 5). Finally, 5 Hz ITC thought to reflect attention to the distractor was not only present during “miss” trials, but also significantly greater during “miss” trials than during “hit” trials, and was negatively correlated with parietal gamma power (Figure 6). Together, these findings converge to provide compelling evidence for increases in parietal gamma power as an index of neuronal processing supporting cognitive control, especially when resisting a distractor.

In previous studies, the enhancement of local gamma power was most robustly reported during the attentional selection of sensory information [31,66,67,68]. Moreover, human EEG, magnetoencephalography (MEG), and intracranial EEG (iEEG) studies repetitively demonstrated that gamma-band oscillation is increased for attended compared to unattended or ignored stimuli in the visual [69,70], auditory [71,72], and the somatosensory cortex [73,74]. In contrast, local gamma power in the higher association areas such as frontal and parietal regions has not been as extensively studied. Increased parietal gamma has been observed during the pre-saccade period in a delayed saccade task and interpreted as encoding the motor goals in the visuomotor processing for saccades [75,76]. This study highlights that gamma enhancements during distractor-evoked conditions are important for attentional control, and therefore, may be reflective of a top–down modulatory mechanism in higher association areas.

Our methodological shift towards a more liberal bias during distraction contrasts with our previous studies employing flashing-screen dSAT with humans [25,26,27,64]. In those prior studies, participants typically became more, rather than less, conservative when the distractor was introduced. This discrepancy may be attributed to differences in distractor implementation. In prior studies, rapid contrast changes in the entire background likely heightened noise and perceptual difficulty, leading participants to adopt a more conservative approach due to decreased signal visibility. Conversely, in the current study, the distractor comprised distinct, sudden-onset visual stimuli, potentially prompting a more liberal response bias with increased false alarms. While speculative, this explanation underscores the importance of considering the disparity between this and previous studies when interpreting results.

Additionally, there were also some unexpected aspects of the results. Participants who showed greater increases in parietal local gamma power during the distractor condition had increased impairment in detection performance (Figure 4). This finding along with the findings of increased gamma and signal detection sensitivity (Figure 2) may reflect a different role of gamma power than previously considered. These results suggest that increases in gamma power may be more relevant for cue detection rather than for resisting a distractor. This may also reflect gamma’s role in attentional effort when resisting the distractor, suggesting that signal detection may utilize both top–down and bottom–up control [77,78]. Additionally, the findings in Figure 4 become even greater when right prefrontal gamma is controlled for, suggesting that right prefrontal gamma is contributing to the control of the left parietal gamma effect. Based on our previous rodent and fMRI studies [64,65], we had initially expected that right PFC would be the primary locus of our effects; however, it was left parietal. Fully determining the reasons for these differences would likely require a series of experiments, but as a general hypothesis, we suspect that the explanation lies in the difference in how the distractor condition was implemented. In those previous studies, the distractor consisted of a whole-field change in the background contrast, likely increasing the perceptual difficulty of detection. In the present study, the background contrast remained constant across the whole field, but shifted within the field to give the appearance of appearing/disappearing squares and rectangles. These shifts would have the potential to draw attention away from the signal, a suggestion supported by the increase in 5 Hz ITC during distractor conditions, particularly during misses.

Therefore, the critical operations for resisting the distractor in previous studies were most likely those involved in amplifying the representation and detection of the signal, whereas in the present study they would be those involved in keeping attention from being captured by the distractor. This explanation would be consistent with theories that right-lateralized ventral frontoparietal networks are specialized for the detection of relevant stimuli (the process we suggest may have been taxed in the “classic” dSAT), whereas parietal regions are more involved in top–down attention and selection (the processes we suggest may have been taxed by the current distractor), and the left parietal cortex being described as particularly important for integrating stimulus representations with the appropriate task set [2,79]. The role of the visual cortex may also be a key component for resisting the distractor. Attention-demanding distractors influence the sensory information stored in the visual cortex while also increasing visual cortical activity in response to the distractor [80,81]. One emerging theory is that the visual cortex is crucial for maintaining working memory representations despite incoming visual distractors [80,81,82]. Therefore, further investigation may be necessary to understand the importance of the combined roles of the PFC, parietal cortex, and visual cortex for attention and distraction resistance.

The research conducted by Corbetta and Shulman (2002) might help explain the differences in left-versus-right lateralization, with a focus on the distinct roles of the prefrontal and parietal cortex [2]. As noted earlier, in our previous fMRI studies, greater distraction-related activation of right PFC has been related to larger distractor-related performance impairments. Additionally, participants with a genetic polymorphism thought to reduce cholinergic function did not activate right PFC in response to distraction but did not show performance decrements relative to controls, suggesting that right PFC does not contribute directly to the control processes needed to maintain performance [25,65]. In one study conducted by Berry et al. (2015), it was observed that connectivity between the right PFC and anterior cingulate correlated with greater distraction-related performance decrements [25]. Conversely, those with strong connectivity between the right PFC and right parietal cortex were less affected by distractors. This aligns with recent research highlighting the involvement of the anterior cingulate and the medial cingulate cortex as hubs for neuronal oscillatory activity when implicated in top–down mechanisms for cognitive control [83,84]. The right PFC is implicated in heightened cognitive demand, as seen in studies across various cognitive domains [85,86,87,88]. This activation reflects the concept of “attentional effort”, where the right PFC initiates and coordinates with other brain regions, such as the parietal cortex, to efficiently manage heightened cognitive demands across different tasks [22].

In further support of right PFC activation, the right PFC emerged as an important hub in the present study for linking neural activity with behavioral variability. Specifically, individuals who reported greater subjective difficulty due to the distractor were anticipated to exhibit heightened variability in response times, despite maintaining accuracy. This escalation in variance was found to be positively correlated with increases in gamma peak variance within the right PFC. Recent animal studies utilizing basal forebrain cholinergic stimulation (electrical or optogenetic) indicate a reduction in low-frequency power and an increase in high-frequency power [89,90,91]. While these studies did not specifically explore the right PFC, similar principles may elucidate the observed increase in right PFC cholinergic activity in previous studies on the dSAT, along with the gamma variability patterns. The cholinergic innervation of the right PFC may be crucial for sensitivity to the elevated load imposed by the distractor. In contrast, parietal regions, where cholinergic innervation also plays a critical role, may be more instrumental in the implementation of top–down control in response to the increased cognitive load [64,92]. This may be in-part due to the role of the cholinergic system in mediating higher order cognitive processing, including top–down attention [11].

## 5. Conclusions

To summarize, the present study provides novel findings that the local gamma-band power in the left parietal regions reflects a possible top–down attentional control mechanism contributing to distractor resistance. Conversely, variability in the right PFC is related to variability in performance, especially under distraction. These findings align with prior research emphasizing the role of frontoparietal cholinergic innervation in similar tasks [20,21,22,45,64,92,93]. Moreover, these findings build upon previous animal research investigating the cholinergic contributions to gamma coherence and stability, although the connection may not be entirely direct [39,40,94,95,96,97]. The present study also focused on signal detection and distraction, and thus cannot speak to whether the neural mechanisms involved here are specific to those operations or may extend more generally to many situations requiring cognitive control.

## Figures and Tables

**Table 1:** Behavioral results.

	SAT		dSAT	
	m	SD	m	SD
hit rate	0.73	0.12	0.80	0.14
hit response time (ms)	326.01	54.13	374.22	61.20
correct rejection rate	0.96	0.03	0.86	0.09
correct rejection response time (ms)	505.72	61.00	542.53	62.64
d’	2.57	0.70	2.20	0.96
beta	6.00	4.64	1.34	0.95

## Data Availability

The data that support the findings of this study are available on reasonable request from the corresponding author due to ongoing and future analyses with the data set.
